# Effect of intravitreal Bevacizumab on coagulation profile of patients with diabetic retinopathy

**DOI:** 10.12669/pjms.41.2.10289

**Published:** 2025-02

**Authors:** Iqra Khalid, Tayyaba Gul Malik, Hassan Tariq, Usman Sajid

**Affiliations:** 1 Dr. Iqra Khalid PGR Ophthalmology, Eye Unit-I, Lahore General Hospital, Lahore, Pakistan; 2 Prof. Tayyaba Gul Malik Head of Ophthalmology Department, Lahore General Hospital, Lahore, Pakistan; 3 Dr. Hassan Tariq PGR Ophthalmology, Lahore General Hospital, Lahore, Pakistan; 4 Dr. Usman Sajid PGR, Punjab Institute of Cardiology, Lahore, Pakistan

**Keywords:** Anti-vascular endothelial growth factor, Bevacizumab, Diabetic retinopathy, Thromboembolism

## Abstract

**Objectives:**

Intravitreal injections of Bevacizumab are widely used all over the world to treat Diabetic Retinopathy. However, there are thromboembolic events reported with its use. As blood coagulation is related with thromboembolism, the purpose of this study was to determine the effect of intravitreal injection of Bevacizumab on the coagulation profile of diabetic patients.

**Methods:**

It was a quasi-experimental study, conducted at Department of Ophthalmology, Lahore General Hospital from January 2023 to August 2023. Patients with treatment naïve proliferative DR and diabetic macular edema were included and those with renal disease, hypertensive retinopathy, chronic liver disease, ischemic heart disease, previous history of stroke, retinal vascular occlusion, any malignancy, history of using anti coagulants and aspirin and the patients who lost to follow up and did not complete the blood work up after injection were excluded. Patients’ Prothrombin time, bleeding time, clotting time, international normalized ratio and activated partial thromboplastin time were checked before and one week after intravitreal injection of Bevacizumab. Paired t test was used for analysis and p < 0.05 was set for significance.

**Results:**

There were 138 patients who fulfilled the inclusion criteria (92 males and 46 females). The mean age was 55±8 years. There was statistically insignificant difference between the coagulation profile before and after intravitreal injection of Bevacizumab.

**Conclusion:**

Coagulation profile is not affected after one intravitreal injection of Bevacizumab. Other factors must be taken into account while injecting Bevacizumab in patients with history of thromboembolic events.

## INTRODUCTION

Diabetes mellitus (DM) is affecting 415 million individuals round the globe which is expected to reach 642 million by the year 2040.^1^ If not diagnosed and managed in time, it can lead to Diabetic retinopathy (DR) which is a blinding complication of DM. Thus, management requires a comprehensive strategy that should include prevention, early diagnosis and timely treatment.

Nowadays, anti-vascular endothelial growth factors (Anti-VEGF) are widely used to treat diabetic retinopathy (DR) and diabetic macular edema (DME). Among many anti-VEGF available in the market, Bevacizumab is the most commonly used because of its cost effectiveness. Although it is injected within the vitreous cavity but some amount of the drug enters systemic circulation raising the question of its systemic safety.^2^

There are studies which indicate that intravitreal injection of anti-VEGF is associated with a higher mortality rate as compared to the controls.^3^ Research regarding bevacizumab shows 6% higher mortality than the normal individuals.^4^ Similarly, there are reports of systemic accumulation after monthly injections of intravitreal drugs which can result in side-effects even if not directly introduced into circulation.^5^ Among others, thromboembolism has been reported as a possible side effect of anti-VEGF agents.^6^ It is also observed that the patients who already had stroke or cerebrovascular event had six folds higher risk of death if given intravitreal bevacizumab within three months after the stroke.^7^

As coagulation disorders are closely related with thromboembolism, there needs to be a delicate balance between the coagulation process and coagulation inhibition. Coagulation profile has to be properly regulated to prevent thromboembolic events.^8^ Prothrombin time (PT), bleeding time (BT), clotting time (CT), international normalized ratio (INR), activated partial thromboplastin time (APTT), play a role in thrombosis formation and embolism and indicate the total clotting capability of blood. Literature lacks the effect of bevacizumab on these coagulation factors. This study was designed to see the direct effect of Bevacizumab on these factors in the real life situations.

## METHODS

It was a quasi-experimental study, conducted at Department of Ophthalmology, Lahore General Hospital from January 2023 to August 2023. Patients with treatment naïve proliferative DR and diabetic macular edema were included. Study was carried out according to the declaration of Helsinki and informed consent was taken. Patients with renal disease, hypertensive retinopathy, chronic liver disease, ischemic heart disease, previous history of stroke, retinal vascular occlusion, any malignancy, history of using anti coagulants and aspirin and the patients who lost to follow up and did not complete the blood work up after injection were excluded.

### Ethical approval:

The ethics committee of the Lahore General Hospital approved the study, No. AMC/PGMI/LGH/Article/Research No./68/2024, dated: February 17, 2024.

Complete history and ocular examination were performed including visual status, intraocular pressure measurement, slit lamp examination, detailed fundoscopy and OCT macula. Patients’ PT, BT, CT, INR and APTT were checked. For blood work up, samples were drawn half an hour before the first Intravitreal bevacizumab. Intravitreal injection was given under strict aseptic technique in the operation theater. Antibiotic eye drops were prescribed for three days and the patient was called on the next follow up day. After one week, blood samples were again drawn and sent to the lab for analysis of the studied variables. SPSS version 23 was used for statistical analysis and paired t test with p < 0.05 was set for significance of results.

## RESULTS

There were 138 patients who fulfilled the inclusion criteria (92 males and 46 females). The mean age of the patients was 55±8 years. There was statistically insignificant difference among the variables before and after intravitreal injection of Bevacizumab (Table-I).

**Table-I:** Mean pre-injection, post-injection and p-values for platelet count, INR, PT, APTT, BT and CT.

	Mean Pre-injection	Mean post-injection	P value	Normal Range
Platelet count x 10^3^	315.12 ± 80.2	313.9 ± 79.2	0.558	150 X 450
INR	1.0014± 0.011	1.0012± 0.014	0.887	1
PT	10.9058±1.23	11.0942±0.29	0.106	10-13 seconds
APTT	25.9130±1.65	25.9710±0.36	0.688	25-35 seconds
BT	2.83 ± 0.5	2.82 ± 0.5	1.000	2-9 minutes
CT	9±3.54	9±3.11	0.266	8-15 minutes

## DISCUSSION

The results of this study show no statistically significant difference between the pre-injection and post injection levels of PT, CT, BT, INR and APTT one week after injection. After intravitreal injection, anti-VEGF can overcome the blood-retinal barrier and enter the systemic circulation, causing a significant decrease in VEGF serum concentration. Circulating VEGF protect the integrity and patency of vessels. Prolonged anti-VEGF treatment has the potential to increase the risk of thromboembolic events by damaging the vessel endothelium and integrity. However, according to many clinical trials Bevacizumab is shown to be safe and did not increase the risk of thromboembolism.^9^ A detail analysis of the trials showed that the patients with greater risk of thrombosis were excluded making the drug free from this side effect. On the other hand, it is recommended that the patients with history of myocardial infarction and stroke should avoid these injections.

Thrombosis formation starts after an injury to the vessel wall which is followed by platelet adhesion to the injury site and activation of coagulation factors. The possible mechanism of action of anti-VEGF drugs is the endothelial cell dysfunction secondary to decreased VEGF. The underlying matrix is exposed resulting in repair and sequence of events leading to thrombus formation.^10^

INR is calculated to standardize the PT. While APTT and PT are measured together for coagulation profile. If these values are increased, it shows that the patient has bleeding tendency and vice versa. However, after receiving bevacizumab intravitreal injections, various outcomes have been observed. Contrary to our findings, Li and colleagues described that after two hours of normal dose of intravitreal bevacizumab there was a significant decrease in PT. However, there was no effect on APTT.^11^

Some researchers have studied the effect of intravitreal bevacizumab on plasma D-dimers as well, which when increased lead to thrombosis.^12^,^13^ These could not be performed in our patients due to lack of this facility in the vicinity.

The disparity in results can be due to selection bias as patients with hypertension, using anticoagulants, or ischemic or thromboembolic events were excluded. In the real life situations, patients with DR are taking aspirin and have other associated vascular comorbidities. Similarly patients other than DR and requiring Bevacizumab were also not taken into consideration. There is a possibility of later changes in the coagulation profile as we only studied the effect after one week. Another point to consider is that in real life situations, clinical significance is not the same as statistical significance.

In contrast to our sample which excluded patients with comorbidities, another study found that high blood pressure, long duration of diabetes, and more number of injections were risk factors for development of thromboembolism. The researcher did not take into consideration the coagulation factors separately. We studied the effect of only one injection which again is rarely done in real life scenarios where patient with DR receives multiple injections for treatment.^14^

How thrombotic activity is increased after the injection is another area to be searched. One explanation is that inhibition of systemic VEGF leads to decreased nitric oxide levels resulting in thrombotic event.^15^

Data regarding side effects of bevacizumab remain inconclusive owing to the different study designs and contrasting the real life situations. A retrospective study included 1173 patients who were treated with bevacizumab injections. Cerebrovascular accidents were reported in 0.5% and five deaths were reported.^16^ Another database of Medicare claims revealed there was 57% greater risk of hemorrhagic stroke but statistically insignificant difference in the risk of ischemic stroke.^17^

Even if it is considered as a rare side effect of anti-VEGF, it is important to find out and highlight the underlying mechanism. Insignificant change in coagulation profile points toward other factors to consider regarding this side effect. On the other hand, we need to look at these parameters in patients who had thrombotic event after this injection. Concerns regarding bevacizumab is increased due to its off-label use and comparison with ranibizumab shows a higher risk of stroke and cardiovascular events after this drug.^18^-^20^

In another study, the risk of stroke was found to be higher during first two years post injection. This shows that a series of tests on these coagulation factors might be important to prevent the risk of thromboembolism.^21^ This ambiguity is further increased when we find studies indicating even the repeated doses of bevacizumab was safe without any higher risk of stroke.^22^

Varying results from different studies can be attributed to the selection bias in the studies. This again raises the important question whether the risk is increased due to other factors or due to the systemic side effect of bevacizumzb.^23^

### Strengths of this study:

The strengths of this study is that it addresses the concerns about potential thromboembolic risks associated with its use which are reported with its systemic use. We have focused a high-risk group (diabetic patients with proliferative diabetic retinopathy or diabetic macular edema) and excluded patients with pre-existing systemic conditions or anticoagulant use, ensuring robust and targeted findings.

### Limitations:

Limitations of this study are short duration of study, a sample which is not a true representative of DR patients seen in our clinics. Effect of only one injection was studied in our study, although in real life situations multiple injections are needed which can have cumulative effect. This can be studied in further research.

## CONCLUSION

Coagulation profile is not affected after one intravitreal injection of Bevacizumab. Other factors must be taken into account while injecting Bevacizumab in patients with history of thromboembolic events.

### Author`s Contribution:

**IK:** Conception and design of the work, data acquisition, analysis, and interpretation, drafting the manuscript.

**TGM:** Conceived, designed and did statistical analysis, drafted the manuscript.

**HT:** Literature search, Conceived and designed the research, Critical review.

**US:** Conceived idea, Critical Review.

All authors have approved the final version and are accountable for integrity of the study.
